# Malignant Hypertension Complicated by Posterior Reversible Encephalopathy Syndrome (PRES)

**DOI:** 10.1155/carm/4095365

**Published:** 2025-10-26

**Authors:** Bassem Alhariri, Gaydaa Ali Ahmed Ali, Arwa Elfatih Mohamed Ali, Abdalrahman Mohammed Mostafa, Muhammad Sharif, Memon Noor Illahi

**Affiliations:** ^1^Department of Medicine, Hamad Medical Corporation, HMGH, Doha, Qatar; ^2^Weill Cornell Medicine-Qatar, Doha, Qatar; ^3^Qatar University Medical College, Doha, Qatar; ^4^Medical Education Department, Hamad Medical Corporation, Doha, Qatar

**Keywords:** malignant hypertension, posterior reversible encephalopathy syndrome, seizure

## Abstract

Clinical signs and symptoms of posterior reversible encephalopathy syndrome (PRES) include headache, altered mental state, vision loss, and seizures. Neuroradiological results exhibiting white matter abnormalities, primarily in the parieto-occipital areas of the brain, are linked to PRES. Hypertensive encephalopathy, eclampsia, renal failure, and immunosuppressive or anticancer treatment are the most common conditions associated with PRES. In the context of increasing renovascular hypertension resulting from bilateral atherosclerotic renal artery stenosis, we present a case with PRES coupled with severe hypertension. The patient's MRI brain showed extensive white matter changes consistent with the syndrome. The pathophysiology of PRES is examined, and the significance of an early diagnosis and course of therapy is underlined.

## 1. Introduction

Posterior reversible encephalopathy syndrome (PRES) is a neurological condition characterized by a rapid onset of symptoms, a range of neurological symptoms and distinctive neuroimaging characteristics [1].

The most frequently observed clinical manifestations involve headache, changes in consciousness, and behavior spanning from drowsiness to a state of impaired responsiveness, seizures, nausea, cognitive abnormalities such as confusion and reduced spontaneity in speech, as well as distortions in visual perception [2].

Both clinical and imaging features typically indicate reversibility. Severe complications such as status epilepticus, cerebral ischemia, intracerebral hemorrhage, or intracranial hypertension necessitate intensive care management for about 40% of PRES patients. If the clinical syndrome is immediately diagnosed and treated, its symptoms usually subside within 1 week, and the changes recorded on magnetic resonance imaging (MRI) disappear during the course of a few days to weeks. PRES syndrome is linked to conditions such as hypertension, as well as vascular and autoimmune diseases, immunosuppressive therapy, and organ transplants. It was noted in a variety of disorders associated with findings on neuroimaging that suggest white-matter edema, mostly in the posterior parietal–temporal–occipital regions of the brain [3].

The syndrome was first recognized in 1996 by Hinchey et al., who reported a group of 15 patients with symptoms such as headaches, seizures, visual impairments, and other specific neurological deficits. Radiological evaluations using computed tomography (CT) or MRI primarily detected cerebral edema in the posterior regions [3].

Since then, PRES has been further detailed through numerous case reports, series, and retrospective observational studies. However, the lack of randomized controlled trials highlights the need for careful interpretation of epidemiological data, diagnostic criteria, and treatment guidelines.

## 2. Presentation

A 43-year-old male with a background of resistant hypertension of 3 years' duration, medication noncompliance, cystitis cystica, and smoking, presented to the Emergency Room with severe bitemporal headache, blurring of vision, and dizziness for 3 days. Patient reported that he stopped taking his antihypertensive medication 3 months ago, had two previous visits to the Emergency with headache, elevated BP, and hypokalemia during the last 3 years, during which he had a thorough workup for secondary hypertension which all came up negative. Previous workup for secondary hypertension, including aldosterone, renin levels, and their ratio, was unremarkable, ruling out primary hyperaldosteronism. Liddle syndrome was considered unlikely in the absence of a strong family history and a normal previous genetic screening panel. His family history is significant for hypertension in his father.

At this presentation, the patient was found to have an elevated blood pressure of 225/141 mm Hg, heart rate of 77 beats per minute, and oxygen saturation of 97% on room air. Fundoscopic examination revealed Grade IV hypertensive retinopathy with papilledema. On neurological examination, he had no focal neurological deficit, GCS 15/15, no cerebellar signs, and muscle power of 5/5 across all limbs.

Significant laboratory results were high creatinine of 132 μmol/L, low potassium of 3.2 mmol/L, low magnesium of 0.6 mmol/L (0.7–1.0), and high glucose of 6.1 mmol/L. Other labs were within normal limits including complete blood count, other electrolytes, troponin, and liver function test.

CT head showed no intracranial bleeding or major vascular territory brain infarction and showed features suggestive of chronic white matter microvascular ischemic changes. Nifedipine and valsartan oral tab were given in the emergency department. After treatment, his blood pressure remained high 219/148 mm Hg, and hydralazine and oral potassium and magnesium supplement were added.

Blood pressure was still on the higher side 201/134 mm Hg, so labetalol infusion started.

On the second day, the patient was admitted to the high dependency unit (HDU) for blood pressure control, where he experienced 2 generalized tonic-clonic seizures within 15 min of each other, without regaining full consciousness in between, consistent with status epilepticus. Systolic blood pressure before seizure was > 200 mm Hg. Lorazepam 2 mg and levetiracetam 3xg IV loading dose were given. This was followed by a decline in mental status demonstrated by confusion, and inability to answer simple questions, GCS 13/15. The patient was shifted to intensive care unit (ICU), and an MRI brain was arranged to rule out PRES syndrome, along with an MR angiogram (MRA) to evaluate reversible cerebral vasoconstriction syndrome (RCVS).

**Figure 1:**
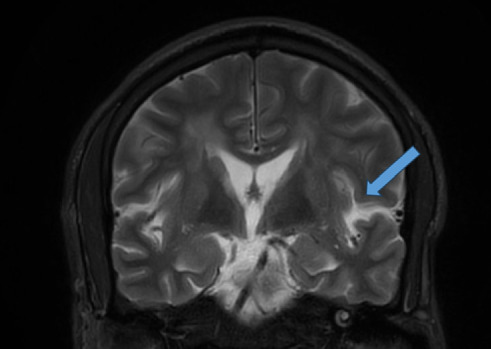
Head MRI. Coronal T2-weighted image demonstrates a focus of increased signal intensity in the right frontal operculum (blue arrow). Axial FLAIR image demonstrates extensive patches of increased signal intensity in the bilateral parieto-occipital subcortical white matter. Axial diffusion-weighted imaging (DWI) shows no restricted diffusion in the corresponding areas, consistent with vasogenic edema.

MRI showed extensive patches of increased signal intensity on T2, and fluid-attenuated inversion recovery (FLAIR) images, seen along the left cerebellar hemisphere and the bilateral periventricular and subcortical regions of both hemispheres, predominantly posteriorly. No obvious areas of restricted diffusion were noted on DWI/ADC sequences. Postcontrast T1-weighted images show left parieto-occipital leptomeningeal enhancement. MRA of the intracranial vessels showed no evidence of vasospasm to suggest RCVS (Figure 1).

On the third day of admission, glyceryl trinitrate infusion was added as blood pressure remained high 180/90 mm Hg. Lumbar puncture performed to rule out meningitis as per MRI findings and CSF were normal, which came back negative for meningitis with normal CSF glucose, protein, and cell levels (Table 1).

The patient started to improve over the following 3 days; blood pressure was controlled, with systolic blood pressure ranging between 160 and 175 mm Hg, and mental status also improved, with a GCS of 14/15; the patient was able to answer questions.

On the sixth day of admission, the patient stepped down to HDU.

Two days later, the patient started to get confused again, was unable to answer simple questions, and was inattentive. Blood pressure was controlled 135/79 mm Hg. An EEG was performed and showed generalized slowing, consistent with encephalopathy but no epileptiform discharges. MRI brain was performed and DWI sequences showed minute foci of diffusion restriction in the left thalamus, left occipital lobe, right frontal subcortical region, and right cortical parietal gray matter.

The following days, the patient's mental status improved with GCS 15/15, blood pressure within normal range, and antiepileptic medication continued.

The patient was discharged after 11 days in the hospital, improved and stable.

## 3. Discussion

PRES syndrome was initially described by Hinchey et al. in 1996 [3]. It has been documented to occur across all age groups, spanning from infancy to elderly individuals, though it tends to be more prevalent among young or middle-aged adults [4].

Conditions related to PRES syndrome include arterial hypertension which is a well-studied cause, and it is the established risk factor in our case [3]. Other risk factors are immunosuppressive therapy, particularly with drugs such as cyclosporine and tacrolimus, renal disease, and pre-eclampsia [5].

The pathogenesis of PRES involves endothelial activation and injury, immune system activation, and release of cytokines. The leading theory, known as the “vasogenic theory,” suggests that rapidly developing hypertension leads to breakdown of the blood–brain barrier and secondary vasogenic edema. This theory is supported by the preferential involvement of the posterior part of the brain due to relative lack of sympathetic innervation [5],which aligns with the finding of our case MRI (extensive patches of increased signal intensity on T2 and FLAIR images along the left cerebellar hemisphere, bilateral periventricular and subcortical regions along both hemispheres predominantly posteriorly). Moreover, prompt management of hypertension often leads to the resolution of symptoms and the reduction of edema seen on radiological images [6], as experienced in our case who improved after his blood pressure was controlled. However, it does not fully explain cases in patients with borderline hypertension or normotension. Other theories, such as the “neuropeptide theory,” propose that release of vasoconstrictors causes vasospasm and cerebral edema. Additionally, cytotoxic and immunogenic theories suggest primary insults from endogenous or exogenous toxins and T-cell activation, respectively. Recent research also suggests involvement of the arginine vasopressin axis in PRES development [5].

The clinical features of PRES often present acutely or subacutely, with symptoms such as encephalopathy, seizures, visual disturbances, headache, and focal neurological deficits. Encephalopathy and new onset seizures are particularly common [7], with varying grades of severity observed. PRES presenting as isolated headache is also reported [8, 9]. Our patient's initial presentation with visual blurring and headache, prior to the development of overt encephalopathy and seizures, aligns with this spectrum. Seizures, including generalized tonic-clonic seizures, partial seizures, and status epilepticus, commonly occur early in the disease course. Despite the high frequency of seizures during the acute phase, the long-term risk of unprovoked seizures is infrequent. Headache, though reported in half of the patients, is usually dull and diffuse. Visual symptoms and focal neurological deficits are also observed in a considerable proportion of patients. Additionally, uncommon manifestations such as myelopathic symptoms, abulia, and ocular apraxia may occur in rare cases [5]. This case presentation followed the pattern of the most common symptoms which is new onset tonic-clonic seizure and headache.

Neurodiagnostic evaluation plays a crucial role in diagnosing and understanding PRES. Serological abnormalities, such as deranged electrolytes, and elevated lactate dehydrogenase levels have been observed in patients with PRES, reflecting endothelial dysfunction [5]. Our patient had low serum potassium level of 3.3 mmol/L, low magnesium, and elevated creatinine of 133 mmol/L (Table 2). The hypokalemia and hypomagnesemia, in the context of metabolic alkalosis (elevated bicarbonate of 30 mmol/L), suggest possible renal potassium wasting, though urinary electrolyte studies were not performed. Hypomagnesemia itself is a recognized contributor to both seizures and PRES [10]. Additionally, elevated C-reactive protein levels have been associated with increased mortality in PRES patients [5], and decreased serum albumin level was also reported in 70% of patients [11] (our patient had a serum albumin of 29 gm/L) (Table 2). The cause of hypoalbuminemia in this case is likely multifactorial, potentially related to poor nutritional status, chronic inflammation, or renal loss, though significant proteinuria was not documented and there was no evidence of chronic liver disease. Cerebrospinal fluid analysis often reveals elevated protein levels [12] (which was within the normal limit in this patient) (Table 1), correlating with the extent and distribution of cerebral edema. Electroencephalogram (EEG) findings vary widely, with patterns ranging from generalized slowing to focal epileptiform discharges, providing valuable information for diagnosis and prognosis. Neuroimaging, particularly MRI, is crucial for confirming PRES diagnosis, with T2-weighted and FLAIR sequences being sensitive for detecting vasogenic edema. Various imaging patterns have been described, including central PRES and atypical presentations such as unilateral involvement or restricted diffusion; in this case, MRI findings were consistent with this classical finding. MRI spectroscopy and perfusion studies further aid in differentiating PRES from other conditions [5]. Overall, a comprehensive neurodiagnostic approach is essential for accurate diagnosis and management of PRES.

The management of PRES relies on supportive care and addressing the underlying triggers [13], although no randomized trials have been conducted to guide specific interventions. Key steps involve removing or reducing the precipitating factors, supportive measures such as hydration and electrolyte correction, including magnesium repletion, monitoring airway and ventilation, and considering prompt delivery in pregnant women or dialysis in renal failure cases [5]. Gradual reduction of acute hypertension is crucial, and treatment of status epilepticus may involve intravenous anticonvulsants or continuous EEG monitoring. Antiepileptic drugs are commonly used during hospitalization (levetiracetam was used to control seizure in our case), but the optimal duration remains uncertain, with a benign long-term prognosis for seizures in PRES patients. Discontinuation of antiepileptic agents after resolution of PRES should be considered once risk factors are controlled [5]. Prompt blood pressure control along with antiepileptic medication and electrolyte correction were sufficient for symptom resolution and improvement of mental status of our patient.

While PRES was initially considered reversible, it can lead to mortality in 19% of cases, with 44% experiencing varying degrees of functional impairment [14]. Factors associated with poor outcomes include severe encephalopathy, certain etiologies such as hypertension and hyperglycemia, delayed treatment, and the presence of comorbidities. Residual structural lesions are seen in 40% of cases on follow-up imaging. Although various imaging features correlate with poor outcomes, identifying a single predictor remains challenging [5]. PRES can also lead to various complications including recurrence and malignant forms. Recurrent PRES occurs in about 4% of patients due to factors such as sickle cell crisis or hypertensive emergencies. Malignant PRES, characterized by severe clinical and radiological features, requires aggressive supportive care, including measures to manage intracranial pressure. Despite its severity, aggressive management has led to favorable outcomes in terms of functional recovery [5].

## 4. Conclusion

PRES syndrome is a neurological condition characterized by a rapid onset of symptoms, a range of neurological symptoms and distinctive neuroimaging characteristics, associated with many medical conditions such as malignant hypertension, autoimmune diseases, renal failure, preeclampsia/eclampsia, and cytotoxic drugs.

High clinical suspicion and prompt recognition of these diverse clinical presentations is crucial for accurate diagnosis and appropriate management of PRES, as the treatment relies mainly on treating the underlying condition.

## Figures and Tables

**Table 1:** CSF findings.

Lab value	Result	Normal range
CSF glucose	4.13 mmol/L	2.22–3.89
CSF protein	0.22 gm/L	0.15–0.45
CSF lactic acid	1.7 mmol/L	1.1–2.4
CSF LDH	< 10.0 U/L	

**Table 2:** Lab results.

Lab value	Result	Normal range
WBC	8.1 × 10^3^/μL	4.0–10.1
HGB	15.8 gm/dL	13.0–17.0
Platelet	221 × 10^3^/μL	150–410
Urea	5.1 mmol/L	2.5–7.8
Creatinine	132 μmol/L	62–106
Sodium	139 mmol/L	133–146
Potassium	3.2 mmol/L	3.5–5.3
Glucose	6.1 mmol/L	3.3–5.5
Bicarbonate	30 mmol/L	22–29
Chloride	99 mmol/L	95–108
Adjusted calcium	2.38 mmol/L	2.20–2.60
Albumin	26 gm/L	35–50

## Data Availability

The data that support the findings of this study are available within the article.
